# Neurological Involvement in COVID-19 Among Non-Hospitalized Adolescents and Young Adults

**DOI:** 10.3389/fneur.2022.915712

**Published:** 2022-06-22

**Authors:** Lise Beier Havdal, Lise Lund Berven, Joel Selvakumar, Tonje Stiansen-Sonerud, Truls Michael Leegaard, Trygve Tjade, Henrik Zetterberg, Kaj Blennow, Vegard Bruun Bratholm Wyller

**Affiliations:** ^1^Department of Pediatrics and Adolescent Health, Akershus University Hospital, Lørenskog, Norway; ^2^Institute of Clinical Medicine, University of Oslo, Oslo, Norway; ^3^Department of Clinical Molecular Biology (EpiGen), University of Oslo and Akershus University Hospital, Lørenskog, Norway; ^4^Department of Microbiology and Infection Control, Akershus University Hospital, Lørenskog, Norway; ^5^Fürst Medical Laboratory, Oslo, Norway; ^6^Department of Psychiatry and Neurochemistry, Institute of Neuroscience and Physiology, Sahlgrenska Academy, University of Gothenburg, Gothenburg, Sweden; ^7^Clinical Neurochemistry Laboratory, Sahlgrenska University Hospital, Gothenburg, Sweden; ^8^Department of Neurodegenerative Disease, UCL Institute of Neurology, London, United Kingdom; ^9^UK Dementia Research Institute, London, United Kingdom; ^10^Hong Kong Center for Neurodegenerative Diseases, Hong Kong, China

**Keywords:** COVID-19, post-COVID syndrome, cognitive functions, adolescents, glial fibrillary acidic protein (GFAp), Neurofilament (NF), fatigue

## Abstract

**Introduction:**

Coronavirus disease 2019 (COVID-19) is prevalent among young people, and neurological involvement has been reported. We investigated neurological symptoms, cognitive test results, and biomarkers of brain injury, as well as associations between these variables in non-hospitalized adolescents and young adults with COVID-19.

**Methods:**

This study reports baseline findings from an ongoing observational cohort study of COVID-19 cases and non-COVID controls aged 12–25 years (Clinical Trials ID: NCT04686734). Symptoms were charted using a standardized questionnaire. Cognitive performance was evaluated by applying tests of working memory, verbal learning, delayed recall, and recognition. The brain injury biomarkers, neurofilament light chain (NfL) and glial fibrillary acidic protein (GFAp), were assayed in serum samples using ultrasensitive immunoassays.

**Results:**

A total of 405 COVID-19 cases and 111 non-COVID cases were prospectively included. Serum Nfl and GFAp concentrations were significantly elevated in COVID-19 cases as compared with non-COVID controls (*p* = 0.050 and *p* = 0.014, respectively). The COVID-19 cases reported more fatigue (*p* < 0.001) and post-exertional malaise (PEM) (*p* = 0.001) compared to non-COVID-19 controls. Cognitive test performance and clinical neurological examination did not differ across the two groups. Within the COVID-19 group, there were no associations between symptoms, cognitive test results, and NfL or GFAp levels. However, fatigue and PEM were strongly associated with older age and female sex.

**Conclusions:**

Non-hospitalized adolescents and young adults with COVID-19 reported more fatigue and PEM and had slightly elevated levels of brain injury markers, but showed normal cognitive performance. No associations were found between symptoms, brain injury markers, and cognitive test results, but fatigue and PEM were strongly related to female sex and older age.

## Introduction

The pandemic of Coronavirus Disease 2019 (COVID-19) caused by the severe acute respiratory syndrome coronavirus 2 (SARS-CoV-2) is an unprecedented threat to health and welfare globally. In the early stages of the pandemic, several case studies provided evidence that infected individuals could suffer neurological complications (1–4). There are reports of neurological symptoms being associated with high SARS-CoV-2 antibody levels in cerebrospinal fluid (CSF) (5), and with demyelinating lesions and other abnormal brain MRI findings (6, 7). In addition, neurological and neuropsychological symptoms such as fatigue, memory loss, and “brain fog” have emerged as prevalent and debilitating symptoms in the acute and subacute stages of COVID-19 (8, 9). However, it is not clear to which extent the neurological manifestations described in severe COVID-19 infections are caused by the virus *per se*, or if they more likely should be attributed to more general consequences of severe disease courses (10, 11). Further, it is yet to be established whether mild COVID-19 is associated with neurological involvement and whether the subjective experience of “brain fog,” fatigue, and other neuropsychological symptoms correspond with objectively measurable cognitive deficits.

With the progression of the COVID-19 pandemic, there is growing concern that symptoms can persist after the initial illness, a condition often referred to as “post-COVID syndrome” (12). A wide range of persisting symptoms are reported, including neurological and neuropsychological complaints such as fatigue, post-exertional malaise (PEM), memory and concentration problems, headache, and muscular pain (13, 14). There are theories that post-COVID syndrome is caused by neuroinflammation (15), induced or exacerbated by a combination of mast cell activation, cytokine storm, and activation of the hypothalamic–pituitary–adrenal (HPA) axis linked to the initial COVID-19 infection (16, 17). Thus, a detailed study of neurological aberrations in the subacute stage of the infection may provide theories of post-COVID syndrome development.

Neurofilament light chain (NfL) is a neuronal protein and is considered a specific biomarker for axonal damage regardless of the cause (18), and is released into CSF upon neuronal injury (19, 20). Though details of kinetics and distribution remain unknown, several studies have shown a tight correlation between levels in CSF and blood (serum and plasma) samples (21, 22), making it widely usable as a biomarker for neuroinflammation and degeneration in neurological conditions (23–26), and has also caught interest as a predictor for neurological outcome in intensive care medicine (27, 28). Another established blood biomarker for brain injury is the glial fibrillary acidic protein (GFAp) (29–31), which is known to increase rapidly in both CSF and serum as a response to acute cerebral injury (32–34), signaling astrocytic activation (35). Thus, GFAp is directly linked to the brain's intrinsic inflammatory system.

The aims of the current study were two-fold: (a) to compare neurological/neuropsychological symptoms, cognitive test results, and serum markers of brain injury (NfL/GFAp) across non-hospitalized adolescents and young adults with COVID-19 (COVID-19 cases) and healthy controls (non-COVID-19 controls); (b) to investigate associations between these variables among the COVID-19 cases.

## Methods

### Study Design

The long-term effects of COVID-19 in Adolescents (LoTECA) project is a longitudinal observational cohort study of SARS-CoV-2 positive and negative non-hospitalized adolescents and young adults, with a total follow-up time of 12 months (Clinical Trials ID: NCT04686734). Details of the design are reported elsewhere (36). In this study, results from the baseline visit are reported. The project has been approved by the Norwegian National Committee for Ethics in Medical research. Informed consent was obtained from all participants.

### Participants

From late December 2020 through May 2021, adolescents and young adults were recruited to the LoTECA study. Inclusion criteria for the COVID-19 cases were: (1) age between 12 and 25 years; (2) positive PCR test for SARS-CoV-2. Exclusion criteria were: (1) more than 28 days since the first day of symptoms (for asymptomatic patients, day one of the disease episode was considered the date of the positive PCR test); (2) hospitalization due to COVID-19; (3) pregnancy. Inclusion criteria for the non-COVID-19 controls were: (1) age between 12 and 25 years; (2) negative PCR test for SARS-CoV-2, no older than 28 days. Exclusion criteria were: (1) history of COVID-19 prior to inclusion; (2) pregnancy.

Individuals eligible for inclusion in either of the two groups were identified through lists of individuals tested for SARS-CoV-2 by PCR received from two accredited microbiological laboratories (Fürst Medical Laboratories; Dept. of Microbiology and Infection Control, Akershus University Hospital), serving the counties of Oslo and Viken, Norway. For those who consented to participate, an appointment at the study center at Akershus University Hospital, Norway, was scheduled as soon as possible after the end of their 10-day quarantine period.

### Investigational Program

The investigational program included clinical examination, blood sampling, spirometry, 3-lead ECG monitoring for 5 min at rest, cognitive testing, and questionnaire charting (36). Approximately halfway through the inclusion period, a neurological examination was included in the clinical examination. Only selected variables relevant to the specific aims of the present study are reported here.

#### Laboratory Assays

Blood samples were obtained from antecubital venipuncture and assayed for routine clinical markers. All samples were tested with Elecsys^®^ Anti-SARS-CoV-2 immunoassay (Roche Diagnostics, Cobra e801, Mannheim, Germany) to detect IgG/IgM against SARS-CoV-2 nucleocapsid antigen. Serum samples from some study participants were retested with the Liaison^®^ SARS-CoV-2 S1/S2 IgG immunoassay (DiaSorin, Saluggia, Italy) to quantify antibodies (IgG) against the spike (S)1 and S2 protein of SARS-CoV-2.

Blood for GFAp and NfL measurements in the serum was collected in 3.5 mL Vacuette R (Greiner bio-one GmbH) with gel, allowed to clot for at least 30 min, processed within 2 h by centrifugation (2200 g, 10 min), and aliquots stored immediately at −80°C until analysis. Serum GFAp and NfL measurements were performed at the Clinical Neurochemistry Laboratory, Sahlgrenska University Hospital, Sweden, by board-certified laboratory technicians blinded to clinical data using commercially available Single molecule array (Simoa) assays on an HD-X Analyzer (Human Neuro 2-Plex B assay), as described by the manufacturer (Quanterix, Billerica, MA). Calibrators were run in duplicates, while samples were diluted four-fold and run in singlicates. Two quality control (QC) samples with different levels were run in duplicates at the beginning and the end of each run. Repeatability and intermediate precision were both 8.7% for the QC sample with an NfL concentration of 8.4 pg/mL and 5.9% for the 79.6 pg/mL sample. For GFAP, repeatability was 6.5% and intermediate precision 7.3% for the QC sample at 102 pg/mL, and repeatability was 5.8% and intermediate precision 6.7% for the QC sample at 388 pg/mL.

#### Cognitive Testing

All participants underwent cognitive testing in the form of digit-span test from the Wechsler Intelligence Scale for Children, 4th edition (WISC) (37) and the Hopkins Verbal Learning Test-Revised (HVLT-R) (38). The digit span test is used for verbal and auditory working memory assessment. A string of random digits is read aloud by the examiner. The first string consists of two random numbers, and for every other string, one more number is added. The digit span forward mode requires the test subject to repeat the digits in the same order as they are presented; in the digit span backward mode, digits are repeated in reverse order. Each correctly repeated string is scored one point. The test is discontinued when two strings of equal length are answered incorrectly. Sum scores for digit span forward and backward, as well as total sum score are reported.

In the HVLT-R test of verbal learning, delayed recall, and recognition, the examiner reads aloud a list of 12 words and the participant is asked to repeat as many words as possible in three consecutive trials. Verbal learning memory is the sum score of remembered words (0–36) in the three trials. Delayed verbal memory is measured as the number of words the test subject recalls after 20 min. Finally, 24 words are read aloud, of which 12 are identical to the previous list of words; the number of correctly recognized and falsely recognized words is recorded separately.

#### Questionnaires

The questionnaire contained questions on demographic background information and symptoms during the disease episode. In general, the frequency of specific symptoms was scored on five-point Likert scales (1–5) ranging from never to each day/always. Information on sex and ethnicity was self-reported. In addition, results from the following validated instruments are reported in the current paper:

Chalder Fatigue questionnaire (CFQ) addresses symptoms of mental and physical fatigue. The 11-item version used in this study has been validated as an assessment tool of chronic fatigue syndrome (39). Each item was scored on a four-point Likert scale (0–3), and CFQ was reported with a total range of 0–33.

Five items from DePaul Symptom Questionnaire (40) were used to address post-exertional malaise (PEM). The frequency of symptoms was rated on a five-point Likert scale, each item scored 0–4, ranging from never to each day/always. Scoring across all items was averaged and then multiplied with 25 to obtain a 0–100 scoring range.

Sleep-related problems were assessed using 12 items from the Karolinska Sleep Questionnaire (KSQ) (41), each item scored on a six-point Likert scale. Results were reported as the average score of all items ranging from 1 to 6 (lower scores correspond to more symptoms), as well as sub-scores for insomnia, awakening problems, and sleepiness.

Brief pain inventory (BPI) (42) is a four-item tool scoring pain from no pain to worst pain ever on a ten-point Likert scale. Results are reported as a summary score (ranging from 4–40) as well as the scores on each item.

### Statistical Analysis

For the cross-sectional comparisons across the COVID-19 cases and non-COVID-19 controls, chi-square test, *t*-test, and Wilcoxon rank-sum test were applied as appropriate, depending on distribution. Associations between variables were first explored by the non-parametric statistics Spearman's rho; thereafter, associations between fatigue score and markers of neuronal injury (NfL/GFAp) were assessed by applying linear regression modeling while adjusting for possible demographic confounders (age, sex, and chronic disease).

Statistical analyses were performed using Stata Statistical Software: Release 16 (StataCorp LLC, College Station, TX). A *p* < 0.05 was considered statistically significant (two-sided test); *p-*values were not adjusted for test multiplicity.

## Results

**Figure 1:**
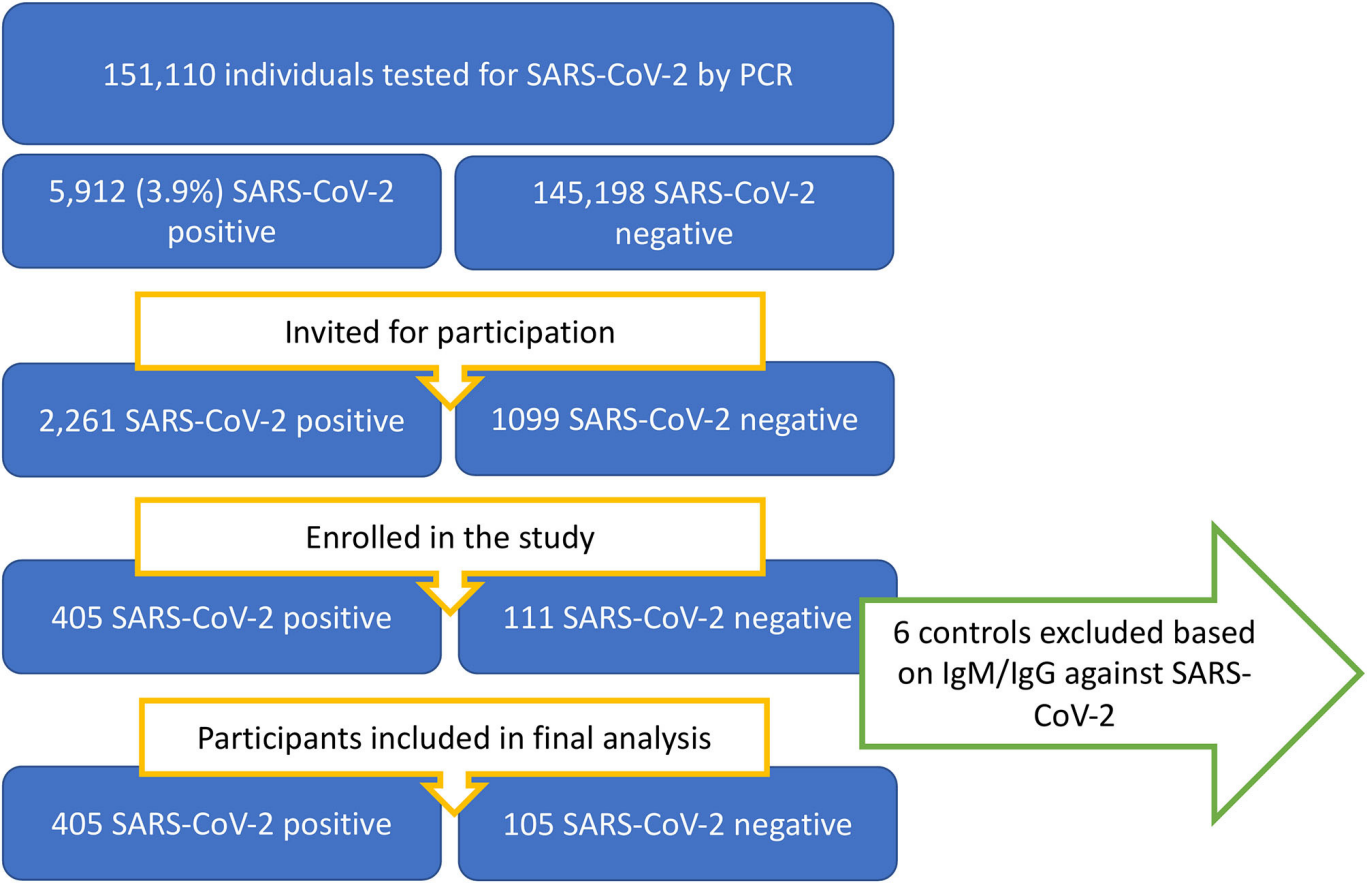
Flowchart of patient availability, identification, and recruitment process.

In the period from 24/12/2020 through 18/05/2021, patients were recruited among individuals between 12 and 25 years of age who had a SARS-CoV-2 PCR test performed at the two collaborating microbiological laboratories. A flowchart of the recruitment proses is presented in Figure 1.

Of all individuals in the background population with a positive SARS-CoV-2 test, 49% were women. Of all SARS-CoV-2 positive cases enrolled, 60% were female. Of individuals younger than 18 years of age, the proportion of recruited participants did not differ between the sexes. For individuals older than 18 years of age, significantly more of the invited women accepted study participation compared to men. The median time from onset of symptoms to enrolment was 18 days.

Sensitivity analysis was performed between models including and excluding six SARS-CoV-2 negative controls who turned out to have IgG/IgM against SARS-CoV-2 nucleocapsid antigen and/or IgG against the spike protein. Their exclusion did not affect the results in the final model.

### Cross-Sectional Comparison of COVID-19 Cases and Non-COVID-19 Controls

Background characteristics of cases and controls are reported in Table 1. There was no difference in demographic variables between the COVID-19 cases and non-COVID-19 controls, except for ethnicity, where Caucasians were overrepresented among controls.

**Table 1:** Baseline characteristics of included children and adolescents by SARS-CoV-2 positivity.

	**COVID-19 cases**	**Non-COVID controls**	***p-*value**
Background	No. = 405	No. = 105	
Female sex, No. (%)	245 (61)	69 (66)	0.34^a^
Age, median(iqr)	17.8 (15.0–21.4)	17.6 (14.7–19.1)	0.08^c^
Days from onset/test, median (iqr)	18 (15–22)	na	
BMI kg/m2, mean (SD)	22.8 (4.5)	22.7 (4.1)	0.87^b^
Ethnicity			
Caucasian, No. (%)	293 (73)	100 (95)	<0.001^a^
Other, No. (%)	106 (26)	4 (4)	<0.001^a^
Chronic disease, self, No. (%)	58 (14)	20 (19)	0.28^a^
Chronic disease, family member, No. (%)	131 (32)	37 (35)	0.70^a^
Highest level of education among parents		
Primary school, No. (%)	3 (1)	0 (0.0)	
Secondary school, No. (%)	71 (20)	14 (14)	
Lower university, No. (%)	180 (49)	57 (59)	
Higher university, No. (%)	111 (30)	26 (27)	0.32^a^
Baseline biomarkers			
SARS-CoV-2-total antibodies, median (iqr)	4.0 (0.9–15.0)	na	–
S-CRP mg/L^d^, No.			
<1	78	308	
1–5	17	61	
>5	4	16	0.950^a^
B-Leukocytes*10^∧^9/L, mean(SD)	5.9 (1.5)	5.6 (1.3)	0.06^b^
B-Platelets*10^∧^9/L, mean(SD)	260 (56.8)	254 (50.9)	0.35^b^
P-Ferritin μg/L, median(iqr)	69.0 (43.0–107.0)	46.5 (31.0–67.0)	<0.001^c^
P-CK U/L, median(iqr)	78.0 (57.0–130.0)	95.0 (69.0–160.0)	0.007^c^
P-INR, median(iqr)	1.1 (0.1)	1.1 (0.1)	0.21^b^
P-D-dimer mg/L, median(iqr)	0.2 (0.1–0.3)	0.1 (0.1–0.2)	0.65^c^
B-Hemoglobin g/dL, mean (SD)	13.5 (1.2)	13.5 (1.1)	0.75^b^
S-Sodium mmol/L, mean(SD)	141 (2.0)	141 (1.7)	0.53^b^
S-Potassium mmol/L, mean(SD)	4.0 (0.3)	4.1 (0.24)	0.10^b^
P-Creatinine, mean(SD)	62.1 (13.3)	61.0 (12.5)	0.49^b^
P-LD U/L, mean(SD)	170 (30)	171 (33)	0.70^b^
P-ALT U/L, median(iqr)	16.0 (11.0–22.0)	15.0 (12.0–20.0)	0.84^c^

a *Chi-square test*;

b *Independent sample t-test*;

c *Wilcoxon rank sum test*;

d *As the majority of participants had S-CRP below lower detection limit (<1), findings are reported as frequency within categories. No finding was above 21 mg/l*.

Comparison of self-reported symptoms showed no difference between the two groups in terms of headache, disorientation, concentration or memory difficulties, sleep, and pain, but the COVID-19 cases scored significantly higher on both fatigue (*p* < 0.001) and PEM (*p* = 0.001). Non-COVID-19 controls reported more difficulties making decisions (Table 2).

**Table 2:** Symptoms, clinical and laboratory findings, and cognitive test results among COVID-19 cases and non-COVID controls.

	**COVID-19 cases**	**Non-COVID controls**	***p-*value^a^**
**Reported symptoms**			
**Fatigue and post-exertional malaise**			
Chalder fatigue score -mean (SD)	16.2 (5.7)	13.5 (4.6)	<0.001
Confidence interval	15.6 to 16.8	12.6 to 14.4	
Post exertional malaise score, -median (iqr)	20.0 (5.0–45.0)	10.0 (5.0–25.0)	0.001
Confidence interval	15 to 25	10 to 15	
**Cognitive symptoms**			
Concentration difficulty, score -mean (SD)	2.6 (1.4)	2.7 (1.1)	0.34
Confidence interval	2.4 to 2.7	2.5 to 2.9	
Difficulty making decisions, score -mean (SD)	1.9 (1.2)	2.2 (1.3)	0.01
Confidence interval	1.7 to 2.0	2.0 to 2.5	
Memory difficulty, score -mean (SD)	1.9 (1.1)	2.1 (1.2)	0.07
Confidence interval	1.8 to 2.0	1.9 to 2.3	
Feeling confused or disoriented, score -mean (SD)	1.4 (0.9)	1.4 (0.7)	0.34
Confidence interval	1.4 to 1.5	1.2 to 1.4	
**Sleep**			
Karolinska sleep questionnaire, -mean (SD)	4.0 (1.2)	3.9 (0.9)	0.21
Confidence interval	3.9 to 4.1	3.7 to 4.1	
*Insomnia, -mean (SD)*	4.2 (1.3)	4.0 (1.1)	0.17
Confidence interval	4.1 to 4.3	3.8 to 4.2	
*Awakening problems, -mean (SD)*	3.7 (1.5)	3.6 (1.2)	0.45
Confidence interval	3.5 to 3.8	3.3 to 3.8	
*Sleepiness, -mean (SD)*	4.1 (1.3)	4.1 (1.0)	0.59
Confidence interval	4.0 to 4.3	3.9 to 4.3	
**Pain**			
Headache, score -mean (SD)	2.5 (1.3)	2.2 (1.1)	0.07
Confidence interval	2.4 to 2.6	2.0 to 2.6	
Brief pain inventory total score, mean (SD)	10.2 (5.4)	10.8 (4.7)	0.29
Confidence interval	9.6 to 10.7	9.8 to 11.7	
*Worst pain in 24 h, mean (SD)*	3.9 (2.3)	4.5 (2.3)	0.02
Confidence interval	3.7 to 4.1	4.0 to 4.9	
*Least pain in 24 hours, mean (SD)*	1.7 (1.4)	1.6 (0.9)	0.28
Confidence interval	1.6 to 1.9	1.4 to 1.7	
*Pain on average, mean (SD)*	2.7 (1.7)	3.0 (1.6)	0.07
Confidence interval	2.5 to 2.9	2.7 to 3.4	
*Pain right now, mean (SD)*	1.8 (1.5)	1.7 (1.2)	0.35
Confidence interval	1.7 to 1.9	1.4 to 1.9	
**Neurological findings and brain injury biomarkers**			
Neurological examination, any findings -No. (%)	6 (3)	1 (1)	0.33^b^
Neurofilament light chain, pg/mL, median (iqr)	4.2 (3.3–5.3)	3.8 (3.1–5.0)	0.05^c^
Confidence interval	4.0 to 4.4	3.6 to 4.1	
Glial fibrillary acidic protein, pg/mL, median (iqr)	61.1 (46.2–77.7)	54.4 (41.0–69.8)	0.01^c^
Confidence interval	58.9 to 64.0	49.1 to 59.2	
**Cognitive test results**			
Digit span forward, total sum score -mean (SD)	5.7 (2.0)	5.6 (1.8)	0.80
Confidence interval	5.5 to 5.9	5.3 to 6.0	
Digit span backward, total sum score -mean (SD)	9.4 (2.2)	9.4 (2.3)	0.84
Confidence interval	9.2 to 9.6	8.9 to 9.8	
Digit span summary score, -mean (SD)	15.1 (3.6)	15.0 (3.4)	0.79
Confidence interval	14.7 to 15.4	14.3 to 15.6	
Hopkins verbal learning test-r evised			
HVLT-R, immediate recall, total sum score -mean (SD)	24.6 (4.2)	24.3 (3.9)	0.44
Confidence interval	24.2 to 25.0	23.5 to 25.0	
HVLT-R, delayed recall, total sum score -mean (SD)	8.7 (2.1)	8.4 (1.9)	0.18
Confidence interval	8.5 to 8.9	8.0 to 8.8	
HVLT-R correct recognition, mean (SD)	11.6 (0.6)	11.6 (0.8)	0.41
Confidence interval	11.6 to 11.7	11.5 to 11.8	
HVLT-R false recognition, mean (SD)	0.3 (0.6)	0.4 (0.8)	0.42
Confidence interval	0.25 to 0.37	0.21 to 0.53	

a *Independent sample t-test unless otherwise stated*;

b *Chi-square test*;

c *Wilcoxon rank-sum test*.

**Figure 2:**
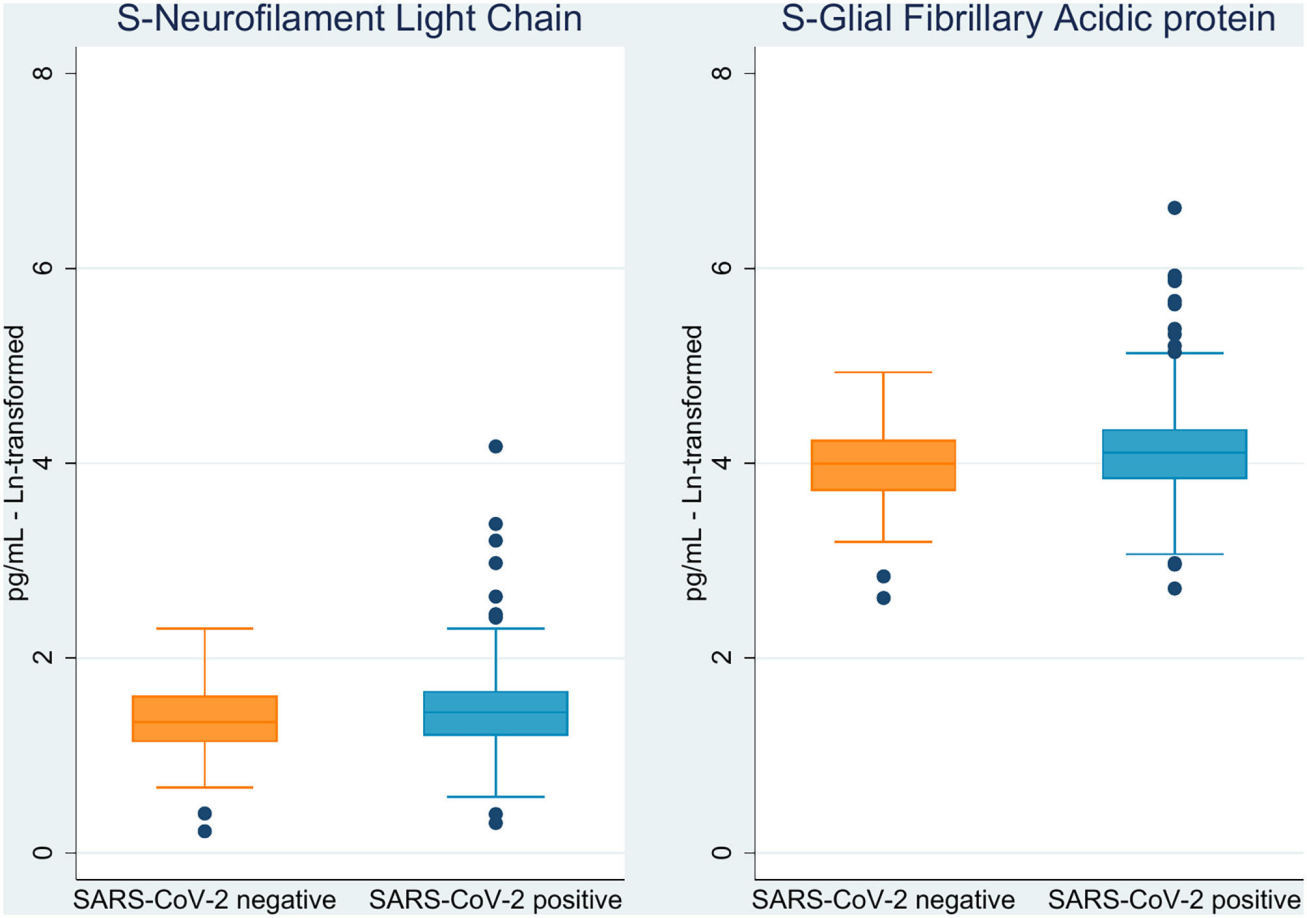
Boxplot of brain injury biomarkers according to SARS-CoV-2 status.

The markers NfL and GFAp were significantly elevated in COVID-19 cases as compared to non-COVID-19 controls (*p* = 0.05 and *p* = 0.01, respectively; Figure 2 and Table 2). Cognitive test results did not differ between the COVID-19 cases and non-COVID-19 controls. As for neurological examination, findings were generally sparse, and no difference was observed between the two groups. Differences in ethnicity among cases and controls did not significantly confound other between-group differences in adjusted analyses.

### Associations to Fatigue Within the SARS-CoV-2 Positive Cohort

Among COVID-19 cases, serum GFAp was negatively correlated with fatigue score, HVLT-R delayed recall, and HVLT-R false recognition, though none of these findings were significant after Bonferroni correction (Table 3). Neither NfL nor GFAp was correlated with any other symptom score, cognitive symptoms, or cognitive test results. Female sex and older age were correlated with all symptom scores for fatigue, sleep, and pain, as well as several cognitive symptoms. Age was associated with cognitive test results. There was no correlation between cognitive test results and reported cognitive symptoms (Table 4).

**Table 3:** Correlation (Spearman's rho) between background variables, symptoms, brain injury markers, and cognitive test results within the COVID-19 group^a^.

		**Sex**	**Age**	**Ethnicity**	**Level of education among parents**	**NfL**	**GFAp**
**Fatigue**
Chalder fatigue score	Corr.coeff. (rho)	0.33	0.31	−0.05	0.01	0.00	−0.10
	*p-*value	**<0.001**	**<0.001**	0.29	0.84	0.93	**0.05**
Post-exertional malaise	Corr.coeff. (rho)	0.21	0.21	−0.03	−0.06	0.01	−0.06
	*p-*value	**<0.001**	**<0.001**	0.50	0.23	0.90	0.22
**Sleep**
KSQ-summary score	Corr.coeff. (rho)	−0.27	−0.19	−0.01	0.04	−0.03	0.03
	*p-*value	**<0.001**	**<0.001**	0.878	0.46	0.56	0.51
**Pain**
Headache	Corr.coeff. (rho)	0.31	0.25	−0.01	0.02	0.03	−0.03
	*p-*value	**<0.001**	**<0.001**	0.91	0.70	0.58	0.58
Brief pain inventory	Corr.coeff. (rho)	0.19	0.08	0.12	−0.17	0.00	−0.04
	*p-*value	**<0.001**	0.10	**0.02**	**0.001**	0.98	0.39
**Cognitive symptoms**
Concentration difficulty	Corr.coeff. (rho)	0.21	0.10	0.01	−0.08	0.03	0.01
	*p-*value	**<0.001**	0.06	0.82	0.15	0.52	0.88
Difficulty making decisions	Corr.coeff. (rho)	0.01	0.13	−0.01	−0.03	0.03	−0.01
	*p-*value	0.91	**0.01**	0.80	0.57	0.60	0.87
Memory difficulty	Corr.coeff. (rho)	0.25	0.10	0.05	−0.13	0.05	0.01
	*p-*value	**<0.001**	**0.05**	0.32	**0.01**	0.31	0.80
Feeling confused or disoriented	Corr.coeff. (rho)	0.15	0.07	0.06	−0.05	0.04	−0.01
	*p-*value	**0.003**	0.18	0.24	0.37	0.40	0.90
**Cognitive test results**
Digit span forward, total sum score	Corr.coeff. (rho)	0.07	0.20	−0.13	0.14	0.00	−0.04
	*p-*value	0.15	**<0.001**	**0.008**	**0.008**	0.93	0.43
Digit span backward, total sum score	Corr.coeff. (rho)	−0.04	0.13	−0.08	0.16	0.02	−0.05
	*p-*value	0.41	**0.008**	0.10	**0.003**	0.74	0.31
HVLT-R, immediate recall, total sum score	Corr.coeff. (rho)	0.03	0.23	−0.20	0.18	−0.01	−0.08
	*p-*value	0.61	**<0.001**	**<0.001**	**0.001**	0.78	0.14
HVLT-R, delayed recall, total sum score	Corr.coeff. (rho)	0.00	0.28	−0.18	0.13	0.08	−0.10
	*p-*value	0.95	**<0.001**	**<0.001**	**0.014**	0.11	**0.04**
HVLT-R correct recognition	Corr.coeff. (rho)	0.04	0.08	−0.09	0.06	−0.02	0.00
	*p-*value	0.47	0.12	0.08	0.24	0.71	>0.99
HVLT-R false recognition	Corr.coeff. (rho)	0.01	0.13	−0.11	−0.01	0.05	−0.10
	*p-*value	0.91	**0.007**	**0.04**	0.92	0.32	**0.04**

*A total of 90 statistical tests are displayed in this table; a Bonferroni-correction for test multiplicity suggests a level of significance at 0.05/90 = 0.0006*.

**Table 4:** Correlation (Spearman's rho) between cognitive symptoms and cognitive test results within the COVID-19 group^a^.

		**Cognitive symptoms**				
**Cognitive test results**		**Concentration difficulty**	**Difficulty making decisions**	**Memory difficulty**	**Feeling confused or disoriented**	**Summary score of cognitive symptoms^b^**
Digit span forward, total sum score	Corr.coeff.(rho)	0.02	0.04	−0.07	−0.08	0.00
	*p-*value	0.64	0.40	0.17	0.14	0.98
Digit span backward, total sum score	Corr.coeff.(rho)	0.03	0.07	−0.05	−0.05	0.02
	*p-*value	0.54	0.15	0.31	0.29	0.70
HVLT-R, immediate recall, total sum score	Corr.coeff.(rho)	−0.10	−0.01	−0.09	−0.09	−0.08
	*p-*value	0.05	0.82	0.09	0.09	0.11
HVLT-R, delayed recall, total sum score	Corr.coeff.(rho)	−0.04	0.06	0.00	−0.03	−0.01
	*p-*value	0.39	0.21	0.99	0.59	0.82
HVLT-R correct recognition	Corr.coeff.(rho)	0.06	0.04	0.03	0.08	0.05
	*p-*value	0.21	0.44	0.57	0.11	0.31
HVLT-R false recognition	Corr.coeff.(rho)	0.02	0.02	0.05	0.02	0.04
	*p-*value	0.63	0.68	0.35	0.63	0.45

a *A total of 30 statistical tests are displayed in this table; a Bonferroni-correction for test multiplicity suggests a level of significance at 0.05/30 = 0.002*.

b *Summary score of cognitive symptoms is an additive score of the four symptom scores; Concentration difficulty, Difficulty making decisions, Memory difficulty and Feeling confused or disorientated*.

In an adjusted linear regression model, there was no association between chalder fatigue score and NfL/GFAp, but fatigue was associated with older age, female sex, and chronic disease (Table 5).

**Table 5:** Association between chalder fatigue score and neuro-injury markers at baseline.

	**Coef (CI 95%)**	***p-*value**		**Coef (CI 95%)**	***p-*value**
NfL^c^ -Ln transformed	−0.52 (−1.84 to 0.80)	0.44	GFAp^d^ -Ln transformed	−0.54 (−1.69 to 0.61)	0.36
Age	0.35 (0.20 to 0.49)	<0.001	Age	0.32 (0.17 to 0.48)	<0.001
Female sex	2.95 (1.83 to 4.06)	<0.001	Female sex	2.97 (1.85 to 4.09)	<0.001
Chronic disease, self	1.64 (0.15 to 3.13)	0.03	Chronic disease, self	1.68 (0.19 to 3.17)	0.03

*Multiple linear regression focusing on NfL^a^ and GFAp^b^ respectively*.

*^a^Neurofilament light chain*;

*^b^Glial fibrillary acidic protein*;

c *R-squared for regression model focusing on NfL = 0.19*;

d *R-squared for regression model focusing on GFAp =0.19*.

## Discussion

The present study of a large group of young, non-hospitalized COVID-19 cases in the late acute stage of the infection show that (a) serum biomarkers of brain injury are slightly elevated, whereas cognitive function tests are normal; (b) fatigue and post-exertional malaise are persistent symptoms, but overall the symptom load was relatively mild; (c) symptoms were not associated with brain injury markers or cognitive tests but correlated with female sex and older age.

The slightly, but significantly increased levels of NfL and GFAp among COVID-19 cases corroborate results from other studies reporting elevation of biomarkers for brain involvement after COVID-19 (43). For instance, Ameres et al. (44) found NfL to be significantly increased in a population of adult health care workers who recently recovered from mild to moderate COVID-19. Also, a small observational study found elevated NfL among severe COVID-19 cases and elevated of GFAp in both moderate and severe cases (45). In a follow-up study prior to the COVID-19 pandemic, elevated levels of NfL in cognitively healthy adults showed an association with the development of mild cognitive impairment (46). In the current study, we found no association between NfL and cognitive test results. The interpretation of NfL results is complicated due to its dependency on age, but since this seems to be a non-linear pattern, and levels are contemplated as quite stable in younger adults (6), we do not think this phenomenon influences our findings.

The absence of between-group differences regarding cognitive test results was surprising, given the frequent report of subjective experiences of cognitive impairment in COVID-19 sufferers. Another study (11) compared cognitive test results in adults recovering from COVID-19 with non-COVID-19 cases and found significantly reduced cognitive performance in the COVID-19 group. However, in this latter study, data were collected from the general population where people were encouraged to answer a questionnaire and/or perform cognitive online, a recruitment procedure vulnerable to selection bias. The psychological stress caused by quarantine, fear, and loneliness will activate stress responses that in turn may influence cognitive capabilities (16, 47–50). It is therefore crucial to compare COVID-19 cases to a matching control population who experienced the same level of social restrictions and other stressors during the pandemic. Even though the proportion of invitees who accepted participation in our study is low, especially among non-COVID-19 controls, we still believe our recruitment procedure is less vulnerable to selection bias compared with the studies reporting contrary results (11, 51).

A previous study of self-reported cognitive symptoms (51) found COVID-19 cases to report significantly more memory problems compared with controls. In contrast, we found no increase in specific cognitive symptoms in the COVID-19 group; in fact, “difficulty making decisions” was significantly (and probably coincidentally) more common among non-COVID-19 controls. However, the COVID-19 group had higher scores for fatigue and post-exertional malaise. Interestingly, these symptoms are a hallmark of post-COVID syndrome as well as persistent symptoms following other infectious diseases (52). The results from the present study may suggest that there is a general tendency for these symptoms to resolve more slowly than other neuropsychological complaints.

We observed a correlation between ethnicity and cognitive test results, and this is suggested to be explained by a strong correlation between parental educational level (proxy of socioeconomic level) and ethnicity.

Interestingly, symptoms were neither associated with serum NfL or GFAp nor with cognitive test results, but did correlate strongly with female sex and older age. This finding endorses previous results from post-COVID syndrome research, where the female sex is consistently reported to be a risk factor, but with limited findings of biological abnormalities (53, 54). The apparent disconnection between clinical symptoms and biological aberrations is an intriguing observation suggesting a biopsychosocial rather than a strict biomedical derivation for the development of persisting symptoms following COVID-19, a perspective deserving further investigations (55).

### Strengths and Limitations

Strengths of the present study include a well-defined group of young individuals undergoing a mild course of COVID-19, recruitment soon after infection, and a comparable control group. A weakness of the study is the higher degree of enrolment among female cases invited to participate in the study, ensuing is a skewness of COVID-19 cases toward more women compared with the background population.

There could be a bias of classification between COVID-19 cases and controls. The COVID-19 controls were recruited among individuals who had a negative PCR test for SARS-CoV-2. These individuals most likely had the PCR test performed either because they had symptoms consistent with COVID-19 or because they had a history of exposure. COVID-19 controls were excluded from the analysis if they had antibodies against SARS-CoV-2. There could still be participants who had a false negative SARS-CoV-2 PCR test and had not undergone seroconversion.

The study is further limited by the delayed implementation of neurological examination in the investigational program. This was not implemented until approximately halfway through the inclusion period, and consequently, this clinical information is not available to all participants. It would have been beneficial if the baseline examination had been completed even earlier in the course of the infection to give a better picture of the acute findings following mild disease. However, the delay in sampling serum for NfL analysis might give a more trustworthy picture, as the rise in serum values following neuronal damage will peak weeks to months after the event (56).

## Conclusion

Non-hospitalized adolescents and young adults in the early convalescent stage of COVID-19 showed no difference in cognitive test results compared with healthy controls, even though blood biomarkers for astrocytic activation and neuronal injury were slightly elevated. Fatigue and post-exertional malaise were more prevalent among the COVID-19 cases but did not correlate with the brain injury markers serum NfL or GFAp nor cognitive test results; however, both symptoms correlated with female sex and older age.

## Data Availability Statement

The datasets presented in this article are not all readily available because of data protection laws. Requests to access the datasets should be directed to corresponding author.

## Ethics Statement

The studies involving human participants were reviewed and approved by Regional Comittee for Medical Research Ethics South East Norway. Written informed consent to participate in this study was provided by the participants' legal guardian/next of kin.

## Author Contributions

VW drafted the study protocol and coordinated the study. LH, LB, JS, TS-S, TL, TT, KB, and HZ contributed directly to the acquisition of data. LH conducted the statistical analysis. LH drafted the manuscript with VW. All authors contributed to the interpretation of the results, revision of the manuscript, and approved the final version for submission.

## Funding

This work was supported by the Norwegian Research Council (grant #302079). HZ is a Wallenberg Scholar supported by grants from the Swedish Research Council (#2018-02532), the European Research Council (#681712), Swedish State Support for Clinical Research (#ALFGBG-720931), the Alzheimer's Drug Discovery Foundation (ADDF), USA (#201809-2016862), the AD Strategic Fund and the Alzheimer's Association (#ADSF-21-831376-C, #ADSF-21-831381-C, and #ADSF-21-831377-C), the Olav Thon Foundation, the Erling-Persson Family Foundation, Stiftelsen för Gamla Tjänarinnor, Hjärnfonden, Sweden (#FO2019-0228), the European Union's Horizon 2020 research and innovation program under the Marie Skłodowska-Curie grant agreement No. 860197 (MIRIADE), European Union Joint Program for Neurodegenerative Disorders (JPND2021-00694), and the UK Dementia Research Institute at UCL. KB is supported by the Swedish Research Council (#2017-00915), the Alzheimer's Drug Discovery Foundation (ADDF), USA (#RDAPB-201809-2016615), the Swedish Alzheimer's Foundation (#AF-930351, #AF-939721, and #AF-968270), Hjärnfonden, Sweden (#FO2017-0243 and #ALZ2022-0006), the Swedish state under the agreement between the Swedish government and the County Councils, the ALF-agreement (#ALFGBG-715986 and #ALFGBG-965240), the European Union Joint Program for Neurodegenerative Disorders (JPND2019-466-236), the National Institute of Health (NIH), USA, (grant #1R01AG068398-01), and the Alzheimer's Association 2021 Zenith Award (ZEN-21-848495).

## Conflict of Interest

The authors declare that the research was conducted in the absence of any commercial or financial relationships that could be construed as a potential conflict of interest.

## Publisher's Note

All claims expressed in this article are solely those of the authors and do not necessarily represent those of their affiliated organizations, or those of the publisher, the editors and the reviewers. Any product that may be evaluated in this article, or claim that may be made by its manufacturer, is not guaranteed or endorsed by the publisher.
